# NETSseq unveils deep molecular insights into astrocyte function in Alzheimer's disease in the human brain

**DOI:** 10.1002/alz70855_106433

**Published:** 2025-12-24

**Authors:** Xiao Xu, Chris Ugbode, Gonca Bayraktar, Marina Lizio, Dino Ossola, Daniel Barker, Justin Powell, David Cadwalladr, Toni Cheung, Jason Lawrence, Keith Page, Craig Thompson, Mark Carlton, Nicola Brice, Lee A Dawson

**Affiliations:** ^1^ Cerevance Ltd, Cambridge, Cambridgeshire, United Kingdom; ^2^ Cerevance, Cambridge, Cambridgeshire, United Kingdom; ^3^ Cerevance, San Diego, CA, USA

## Abstract

**Background:**

Sporadic Alzheimer's disease (AD) is a complex and heterogenous condition, posing challenges for the development of effective therapeutics. A detailed molecular understanding of how specific brain cell types change throughout disease progression can help unravel this complexity, revealing common mechanisms of disease pathophysiology and identifying potential targets for therapeutic intervention.

**Method:**

Cerevance's proprietary technology platform, NETSseq, enables deep gene expression (>12,000 genes detected) and paired epigenetic profiling of specific cell types from post‐mortem human brain By applying NETSseq to brain tissue from control, early‐, and late‐sporadic AD donors, a comprehensive dataset consisting of diverse neuronal and glial cell type profiles has been generated, including a set of astrocyte RNA‐seq and ATAC‐seq samples.

**Result:**

Correlative analysis of paired RNA‐seq and ATAC‐seq data enables the identification of putative genome regulatory regions and their corresponding regulated genes within each cell type. Analysis of control and AD astrocyte samples has revealed numerous chromatin and gene expression changes associated with disease progression, with further analysis revealing the interplay between these changes. Finally, linking these paired datasets with published GWAS AD data, both reproduces previously identified genetic associations, and reveals novel, genetically validated targets with astrocyte‐specific mechanisms of action.

**Conclusion:**

Along with generating highly reproducible molecular profiles from specific CNS cell types, NETSseq enables the understanding of temporal dynamics of chromatin and gene expression changes across disease progression, thereby identifying relevant pathways and genes to target as novel therapeutic strategies.

